# Rabies Infection: An Overview of Lyssavirus-Host Protein Interactions

**DOI:** 10.52547/ibj.25.4.226

**Published:** 2021-06-21

**Authors:** Fatemeh Zandi, Fatemeh Goshadrou, Anna Meyfour, Behrouz Vaziri

**Affiliations:** 1 Medical Biotechnology Department, Biotechnology Research Center, Pasteur Institute of Iran, Tehran, Iran;; 2 Department of Basic Sciences, Faculty of Paramedical Sciences, Shahid Beheshti University of Medical Sciences, Tehran, Iran;; 3 Basic and Molecular Epidemiology of Gastrointestinal Disorders Research Center, Research Institute for Gastroenterology and Liver Diseases, Shahid Beheshti University of Medical Sciences, Tehran, Iran;; 4 Department of Molecular Systems Biology, Cell Science Research Center, Royan Institute for Stem Cell Biology and Technology, Tehran, Iran

**Keywords:** Host-Pathogen Interactions, Lyssavirus, Rabies

## Abstract

Viruses are obligatory intracellular parasites that use cell proteins to take the control of the cell functions in order to accomplish their life cycle. Studying the viral-host interactions would increase our knowledge of the viral biology and mechanisms of pathogenesis. Studies on pathogenesis mechanisms of lyssaviruses, which are the causative agents of rabies, have revealed some important host protein partners for viral proteins, especially for most studied species, i.e. RABV. In this review article, the key physical lyssavirus-host protein interactions, their contributions to rabies infection, and their exploitation are discussed to improve the knowledge about rabies pathogenesis.

## INTRODUCTION

Lyssavirus, a genus of the family *Rhabdoviridae* from order *Mononegavirales*, consists of single-stranded, negative-sense RNA viruses, which infect mammals and cause fatal acute viral encephalomyelitis known as rabies^[^^1^^]^. There are high levels of nucleotide and amino acid sequence homology among lyssaviruses; therefore, illnesses caused by RABV and other lyssaviruses are virtually indistinguishable^[^^2^^]^. RABV, the prototype virus of the genus Lyssavirus, is more globally distributed and abundant with more known progenies amongst species in the genus^[^^1^^]^. Unfortunately, there is no effective therapy for rabies once the symptoms of clinical disease occur. Annually, about 60,000 human rabies deaths are reported worldwide^[^^3^^]^. 

 The ~12 kb RNA genome of lyssaviruses comprised of five genes and encodes viral structural proteins: N (58-62 kDa), P (35-40 kDa), M (22-25 kDa), G (65-80 kDa), and L (190 kDa)^[^^4^^]^. Since N is the most conserved gene in the lyssaviruses genome, it is commonly used for species discrimination in the genus lyssavirus^[^^5^^]^ with 80-82% cut-off value in nucleotide identity^[^^6^^]^. Indeed, the nucleotide identity of 82-100% for N gene could be observed within the species^[^^7^^]^. Based on the updated classification and taxonomy approved and ratified by the International Committee of taxonomy of Viruses in March 2020, the lyssavirus genus is composed of 17 species. There is also an introduced putative lyssavirus that does not yet have taxonomic status^[^^7^^]^. 

 In the virion of lyssaviruses, genomic RNA is tightly encapsidated by N to form helical RNP core together with P and L. Subsequently, the RNP core is surrounded by M, which has a critical role in the virion morphogenesis. M is responsible for the condensation of RNP into the typical bullet-shaped virus particle. This form of morphogenesis plays a pivotal role in the successfulness of assembly, budding, and infectivity of virus since in the absence of M, poor release of rod or round-shaped particles with highly affected infectivity is observed^[^^8^^]^. The RNP-M structure is then surrounded by a host cell-derived lipid bilayer envelope, which contains the surface trimeric Gs (reviewed in^[^^9^^]^). 

**Fig. 1:**
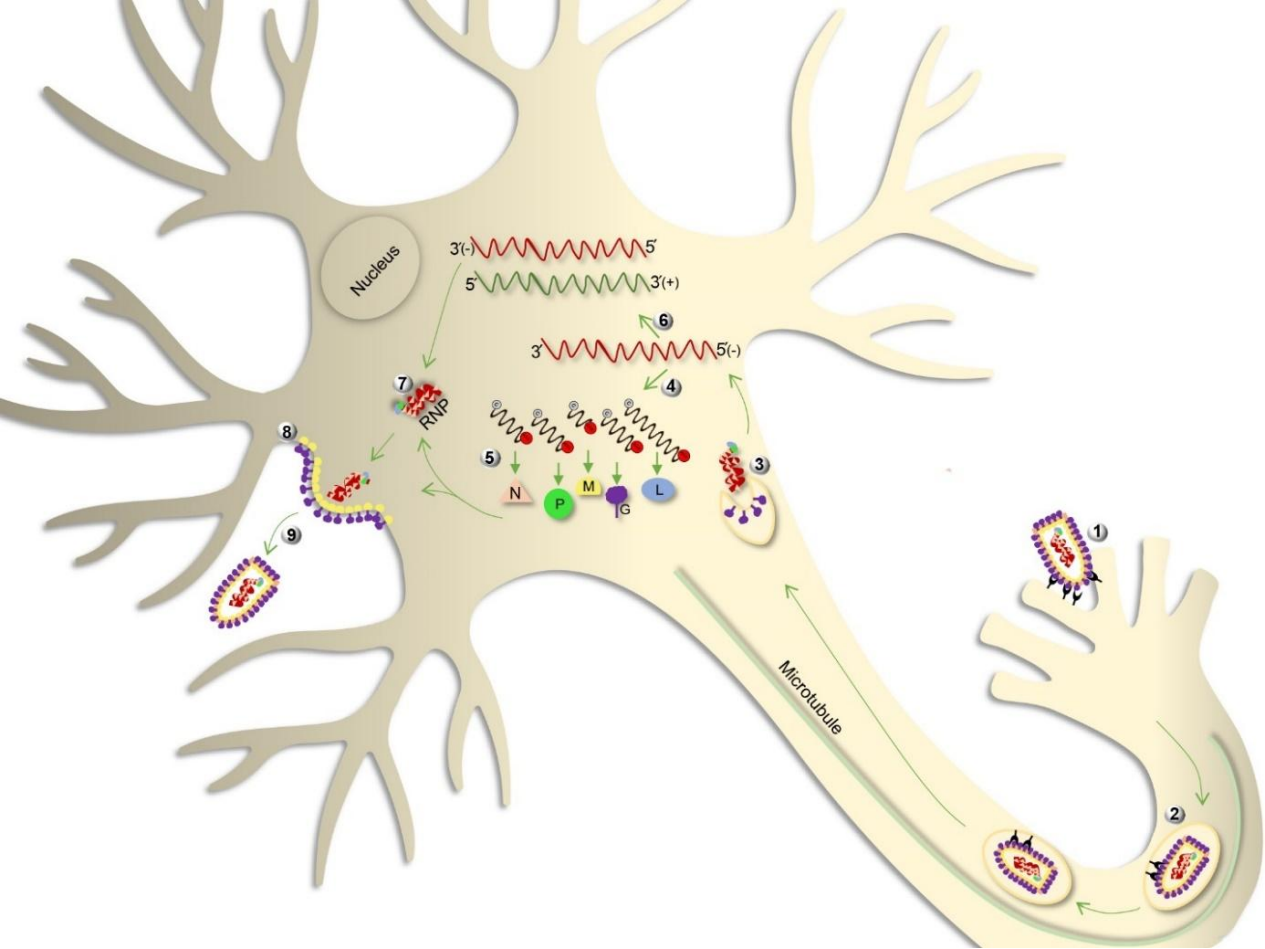
Lyssavirus life cycle in the neuron. Following the attachment of G protein to the neuronal receptors, virus enters the cell through endocytosis (step 1). Virion is then transported within the vesicle along axonal MTs (step 2) and is uncoated in the cell body, releasing the RNP complex (step 3). The encapsidated RNA genome is transcribed to form five mRNAs related to structural genes (step 4), which are then translated into the viral proteins, namely N, P, M, G, and L (step 5). The genome is also transcribed to form the anti-genome, which is a positive-strand intermediate RNA and is used as a template for the replication of genome (step 6). Subsequently, assembly of the viral components (step 7), budding (step 8), and release (step 9) of the virion are achieved

 To start infection, virus attaches to surface cellular receptors through G and enters cell via the endosomal transport pathway (endocytosis). The low pH value of endosome induces a membrane fusion process, followed by the uncoating virus particle and release of helical RNP in the cytosol. In the next step, transcription of the viral genome by the P-L complex happens to produce five positive-strand monocistronic mRNAs and continues by the translation of five viral proteins. The RNA polymerase activity switches from transcription to replication to produce positive-strand replicative RNA (anti-genome), which is a template to make negative strand RNA genome. The synthesized viral RNA is then packaged along with N-P complex and L to form RNP. Then M is associated with RNP complex to condense RNP and localize it at the cell membrane where G is present. Finally, following the interaction of M-RNP complex with cytoplasmic domain of G, the mature virion acquires its envelope by budding through the host cell membrane (reviewed in^[^^9^^]^). Figure 1 represents the lyssavirus life cycle in a neuronal cell.

 Lyssaviruses infect neurons with no signs of neuronal massive injury or death in the routine postmortem examination of rabid patients. Therefore, it seems that the neuronal dysfunction is the main cause of severe neurological symptoms in rabies^[^^10^^]^. On the other hand, the degeneration of dendrites and axons has also been shown in experimental mouse models of rabies^[^^11^^]^. Although there are considerable improvements in understanding the molecular mechanisms of neuronal dysfunction/injury in rabies, its exact mechanism remained to be clearly understood. Findings of some comparative proteomic studies have shown the altered expression of the majority of cytoskeletal proteins during rabies infection, which could be a strong clue for neuronal dysfunction/injury^[^^12^^-^^18^^]^. Further study on cultured neurons infected with RABV CVS strain demonstrated that RABV P interacted with mitochondrial complex I and increased its activity, resulting in the overexpressed ROS and subsequent neuronal degeneration. This finding is postulated as an important basis for neuronal injury/dysfunction in rabies^[^^19^^]^. Moreover, interaction proteomics, a popular and evolving approach to study viral pathogenesis mechanisms^[^^20^^]^, has clarified the important aspects of lyssavirus infection in recent years. The interactions between lyssavirus and host proteins occur at different steps of viral life cycle to facilitate viral replication and consequential pathogenesis. 

 This review provides an overview on prominent experimentally defined lyssavirus-host physical protein interactions reported to date, which might help to construct predictive pathogenesis model(s) for the genus lyssavirus. Notably, most of available PPI data are related to RABV species, which has been more studied. These PPI data have been retrieved from literature mining and VirHostNet database. Briefly, in order to collect the appropriate literature, multiple related keywords, including lyssavirus, rabies, host, interaction, binding, and association, were used to search related articles in PubMed or Google Scholar. Besides, many virus-host interaction databases, such as VirusMINT, VirusMentha, and VirHostNet, were searched to find physical PPI data for the lyssavirus genus. However, only in the VirHostNet knowledgebase, the related PPIs were available. The PPI data (virus-host) of the *Rhabdoviridae* in VirHostNet database family were downloaded from the family rank section, and data belonging to the lyssavirus genus were extracted. 

Lyssavirus proteins and PPIs

*G protein*

 Lyssavirus G, the surface protein of virion, forms trimeric spikes on the viral particle surface. The N-terminal domain of G extends outwards on the lipid envelope of particle, and the C-terminal domain of G inserts under the virion envelope where it associates with M to produce a complete virus (reviewed in^[^^9^^]^). G is the primer component of lyssavirus infection and has various important roles in viral infection, such as the virus attachment to the specific receptors of neurons^[^^21^^]^, induction of virus-neutralizing antibodies^[^^22^^]^, neuronal survival or apoptosis^[^^23^^]^, and virus release^[^^8^^]^. 

 The lyssaviruses neurotropism and their attachment/ entry to target cells are dependent on the protein interactions between G and neurospecific receptors. At least four receptors have been identified for RABV^[^^9^^]^. nAChR, the first identified binding receptor for RABV^[^^21^^]^, has been shown to co-localize with RABV (CVS) in neuromuscular junctions^[^^24^^,^^25^^]^. The binding inhibition of RABV (CVS) and nAChR using monoclonal antibodies against a G peptide demonstrated the contribution of G to the mentioned interaction^[^^26^^]^. Primary replication of virus in muscle before viral entry into the CNS has been proposed as the functional role of this interaction in RABV infection. Alternatively, nAChR may concentrate virus at sites in proximity to peripheral nerves, which facilitate the viral spread along peripheral nerves to the brain^[^^21^^]^. Thoulouze *et al.*^[^^27^^]^ have proposed NCAM as another receptor for G. They found the presence of NCAM on the surface of RABV-susceptible cell lines and the absence of this receptor on the surface of RABV-resistant cell lines. They also showed that the natural ligand or specific antibodies against NCAM significantly decreased RABV (CVS strain) infection *in vitro*, soluble NCAM could neutralize the infectivity of RABV for susceptible cell lines, and rabies mortality was delayed in NCAM-deficient mice^[^^27^^]^. The p75NTR was identified as another ligand for RABV G through the analysis of a cDNA library prepared from a murine neuroblastoma cell line. This interaction was confirmed by Co-IP of p75NTR with RABV G^[^^28^^]^. Using a reverse binding assay, p75NTR interacted with G of certain lyssavirus species, including RABV (wild-type, CVS, and PV strains) and EBLV-2, while no interaction was detected for other studied species. This observation clearly implies the usage of alternative receptor(s) by different lyssaviruses^[^^29^^]^ and may justify differences in their pathogenicity and neuroinvasiveness pathway^[^^30^^-^^32^^]^. *In vivo* studies have indicated the main distribution of p75NTR in the dorsal horn of spinal cord. Thus, RABV G-p75NTR interaction may play a role in retrograde axonal trafficking of RABV particles in the CNS^[^^33^^]^. However, discrepancies between the expression sites of p75NTR and RABV-infected regions of the brain suggest the existence of additional factor(s) involved in RABV axonal transport, which needs further investigation^[^^34^^]^. mGluR2 has recently been introduced as another G receptor for different strains of RABV and WCBV using a RNAi strategy, Co-IP, and pull-down assay^[^^35^^]^. 

 Apart from participating in viral entrance, G has been demonstrated to have ability to target the neuronal enzymes by its PDZ-BS, which mimics the PDZ domain of neuronal enzymes. Such interfering in infection by the virulent strains of RABV ends with cell survival, while with vaccinal strains ends with neurons death. Caillet-Saguy *et al.*^[^^23^^]^ displayed that the G protein of the virulent strain bound to the PDZ domain of MAST2 and inhibited the controlled phosphorylation of PTEN by MAST2. They revealed that the dephosphorylation of PTEN changed its intracellular localization, stability, and activity, leading to altered neuronal homeostasis and neurosurvival^[^^23^^]^. In one study on the network of RABV gene products implicated in rabies using a systems biomedicine approach, authors proposed that G prompted the hyperactivation of PI3K-AKT signaling through the dephosphorylation and redistribution of PTEN. The consequences of the activation and the downstream signaling of AKT could reduce apoptosis or cell survival^[^^36^^]^. On the other hand, G of the vaccinal strain bound to the PDZ domain of MAST2 and other cellular partners, particularly PTPN4, an anti-apoptotic protein. This interaction suppresses the efficient dephosphorylation of ligand(s) by PTPN4. Therefore, the homeostasis of the infected neuron alters, and apoptosis signaling is triggered^[^^23^^]^. 

**Fig. 2:**
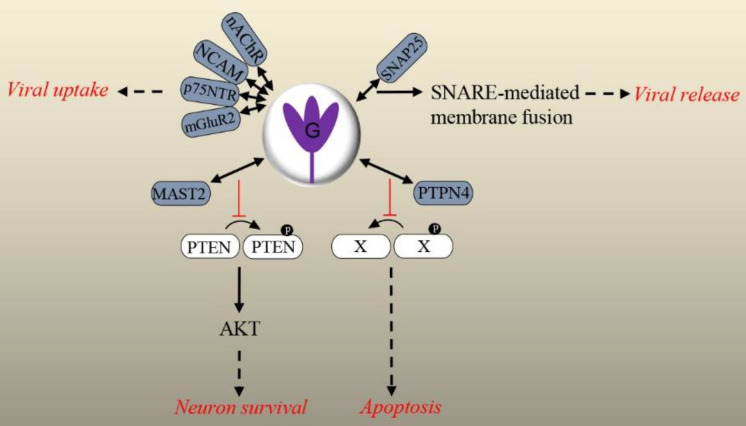
Overview of the validated physical interactions between lyssavirus G and host proteins. Host partners are colored in dark blue. Downstream signaling (if elucidated) and/or functional/pathologic outcomes of PPIs are shown. X represents a cellular ligand, and P in black circle stands for a phosphate. Interaction:  , Stimulation:  , Inhibition:   , Outcome:  .

 G of RABV (CVS-11, SAD strains) also interacts with SNAP25, a member of the SNARE complex that mediates membrane fusion events. Knockdown of SNAP25 showed an inhibitory effect on the release of RABV in nerve cells. It was proposed that the interaction of G and SNAP25 regulated viral release in the nervous system via SNARE complex-mediated membrane fusion. Further research is needed to elucidate the exact mechanism of membrane fusion and progeny virus release^[^^37^^]^. A diagram of the explained G-host PPIs is presented in Figure 2.

 Overall, the promotion of viral virulence, neurosurvival or apoptosis events, and, regulation of viral release are the main consequences of the mentioned G-host protein interactions. Accordingly, G involves in some pathogenetic steps of rabies infection including entry into the nervous system, spread to the CNS, and spread within the CNS via receptor-mediated cell entry.

*N protein*

**Fig. 3:**
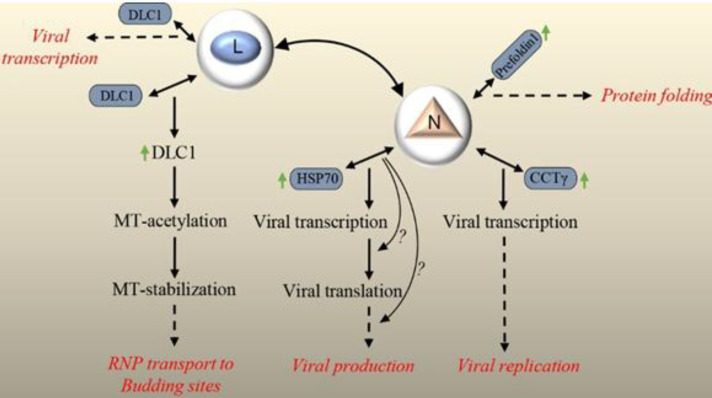
Overview of the validated physical interactions between lyssavirus N, lyssavirus L and host proteins. Host partners are colored in dark blue. Downstream signaling (if elucidated) and/or functional/pathologic outcomes of PPIs are represented. Interaction:  , Stimulation:  , Outcome:  , Upregulation:

 Lyssavirus N, together with the P and L component of the virion-associated RNA polymerase, forms RNP core of the virion, which has RNA polymerase activity. After encapsidation of the viral genome by N, transcription and replication of genome are possible by L^[^^38^^]^. In addition to this basic function, N, as one of the viral proteins, participates in the suppression of the host innate immune response during infection, thus helps virus to replicate and spread efficiently in the brain and CNS. N inhibits the activation of IRF-3 pathway by evading the activation of RIG-I, resulting in the suppression of the expression of host defense-related genes, IFN and chemokines^[^^38^^]^. It has been supposed that the closed form of N-RNA limits the replication of the RNA genome and suppresses the activation of RIG-I; however, the elucidation of involved mechanism(s) needs more efforts. Till now, some few physical partners have been introduced for N. RABV N (CVS strain) binds to Hsp70 chaperone, which positively regulates the RABV infection cycle at different stages, such as the transcriptional and/or translational level and/or viral assembly and budding^[^^39^^]^. Hsp70, as a multifunctional protein, is involved in the cellular processes, including protein translation, folding, trafficking, and degradation^[^^40^^]^. Hsp70 takes part in the replication of numerous viruses^[^^41^^]^. In the case of RABV, upregulation of Hsp70 and its accumulation in NBs, along with its presence in both purified nucleocapsid and virions, have been demonstrated. Downregulation of Hsp70 for elucidating the functional role of N-Hsp70 association using RNAi revealed a decrease in the viral mRNA, proteins, and particles. Reduction of viral protein synthesis and viral production can be the consequences of the affected viral transcription level, but the presence of a specific role for Hsp70 in other steps, including translation and/or assembly and budding, could also be possible. Accordingly, its proviral function during infection and involvement in at least one stage of RABV life cycle are obvious^[^^39^^]^. In a research, the colocalization of RABV N and P (HEP-Flury strain) with neuronal CCTγ and CCTα, two components of the eukaryotic cytosolic chaperone in TRiC/CCT complex involving in protein folding, was indicated in NBs. Knockdown of CCTγ showed the significant inhibition of viral protein expression and replication, which was due to the affected transcription step. Indeed, N in complex with P recruits CCTγ to NBs, which are factories for virus replication and this chaperonin facilitates viral transcription and replication, in general. The detailed mechanism(s) involved in this facilitated replication remains to be clarified^[42]^. In the next study of the same researchers, observation of the colocalization of RABV N (HEP-Flury strain) with prefoldin 1 (a co-chaperone of group II chaperonins) in NBs has been suggested to assist in the folding of viral or host proteins during RABV infection^[^^43^^]^. Prefoldin is an intermediary factor between Hsp70 and TRiC/CCT^[^^44^^]^. The cooperation of this group of proteins is speculated to facilitate RABV replication^[^^43^^]^. Interestingly, the overexpression of Hsp70, CCTγ, and prefoldin 1, together with other chaperonins such as CCTӨ and Hsp90, as molecular chaperones and chaperonins during rabies infection^[^^39^^,^^42^^,^^43^^]^, could be a hint of their cooperation in the folding process of viral proteins and facilitation of RABV replication. A diagram of the explained N-host PPIs is illustrated in Figure 3. 

 Overall, the functional roles of physical association of N with some host factors with crucial activities in protein homeostasis are the positive regulation of viral RNA transcription, translation, protein folding and RNA replication, which are in line with the main activity of RNP complex in rabies infection. 

*L protein*

 Lyssavirus L protein has an RdRp activity. L conducts the transcription of viral mRNAs via its transcriptase, capping, and polyadenylation activity. Also, the replication of the viral genome is conducted by its replicase activity. Interaction of L with its cofactor, P, in RNP core and formation of L-P complex are necessary for viral transcription and replication (reviewed in^[^^9^^]^). The binding of L to N has been suggested to be important for the initiation of genomic RNA synthesis, as well^[^^45^^]^. In order to understand the precise mechanism of the L function, studying its association with host factors would be informative. However, it appears that an intrinsic instability of transiently expressed L is a limiting factor for such studies^[^^46^^]^. Recently, the colocalization of RABV L (SAD B19 strain) with acetylated and reorganized neuronal MTs has been reported, and a DLC1-binding motif in L, similar to that of RABV P has been detected. Mutations of these motifs in L and P demonstrated their involvement in the regulation of DLC1 gene expression and regulation of viral primary transcription. Since RNA polymerase activity of other nonsegmented negative-strand RNA viruses is regulated by cytoskeletal proteins or cytoskeletal-associated proteins, it can be concluded that DLC1, a MAP, acts as a transcription factor for RABV polymerase. However, it has been shown that the regulation of transcription by DLC1 is inessential, and this factor only enhances the primary viral transcription process. Besides, mutations of the DLC1-binding motif in L inhibit MTs acetylation/stabilization and accumulation of L at MTs^[^^47^^]^. According to a previous study^[^^48^^]^, it has been suggested that DLC1 overexpression in RABV infection results in increased acetylation and stabilization of MTs, which the latter has been proposed to stimulate RNP transport to virus budding sites after formation in NBs in cytoplasm^[^^47^^]^. A diagram of the explained L-host PPIs is represented in Figure 3.

 Overall, it is concluded that DLC1-L interaction plays a central role in the RABV infection through the regulation of viral transcription in early steps of infection, regulation of DLC1 gene expression, acetylation/stabilization of MTs, and consequent colocalization of L with acetylated MTs. Precise mechanisms and signaling pathways involved in these events remain to be elucidated. 

*P protein*

P protein of lyssavirus is the co-factor of the viral RNA polymerase with a central role in viral transcription and replication^[9]^. Besides, P functions as a host innate immune antagonist. For P, there are some identified host protein partners, which mostly involve in suppressing the host defense. Subversion of antiviral signaling pathways via these interactions is central to rabies pathogenicity.

P exploits cellular factors to escape immune response via different mechanisms. The IFN-induced PML protein localizes into nuclear bodies with possible functions in nuclear trafficking, apoptosis, and viral defense^[^^49^^]^. The direct interaction of RABV P (CVS strain) C-terminal domain with the RING finger motif of PML leads to the retention of the PML in the cytoplasm. Also, the interaction of P3 isoform of P with PML increases the PML body size in infected cells. P affects the localization and structure of the nuclear bodies and inhibits the antiviral activity of PML, but precise mechanism of this antiviral function is unclear^[^^50^^]^. Inhibition of type I (IFN-α/β) and type II (IFN-γ) IFN-dependent Jak-STAT signaling through the interaction of P from RABV (PV, SAD l16, CVS, and SHBRV strains) and other lyssaviruses (MOKV and ABLV) with the STAT1/2 proteins is a well-described mechanism by which the immune system is sequestered. Type I and II IFN receptors are triggered by related IFNs, and then STATs are phosphorylated by Jak and tyrosine kinase. At this step, P binds to STAT1/2, preventing their translocation to the nucleus and subsequent inhibition of the expression of antiviral products like myxovirus resistance 1, OAS1, and other ISG products. Truncated forms of P can bind directly to the heterodimers of STAT1 and STAT2, which are complexed with IRF9 and form the ISFG3 and also STAT1 homodimers. Finally, the consequence of these bindings is transcriptional inhibition of the ISRE and GAS, respectively. Actually, this inhibitory mechanism represents a key pathogenicity factor in lyssavirus infection^[^^51^^,^^52^^]^. RABV P (CVS) also interacts with activated STAT3 through its C-terminal 30 residues, prevents its nuclear translocation and inhibits the signaling of STAT3^[^^53^^]^, an important mediator of GP130 receptor-dependent signaling pathway. This pathway is activated by other immune molecules, particularly members of the IL-6 cytokine family^[^^54^^]^. Inhibition of the nuclear translocation of GP130-activated STAT3 via P-STAT3 association suppresses the downstream signaling and represents an immune antagonistic role for P beyond IFNs^[^^53^^]^.

The cooperation between P and M proteins of RABV (a street strain:8743THA) to inhibit Jak-STAT signaling has been demonstrated, and an inhibitory model has been proposed. After IFN stimulation, M interacts with Jak1 and then with STAT1 to block their phosphorylation, respectively. Thereafter, M-Jak1 switches to M-STAT1 via P to increase the capacity of P for STAT1 binding and inhibition of STAT dimer translocation to the nucleus. During late infection stages, P and M preferentially bind to Jak1, which might be essential to limit signal outbursts in the early stages of infection and also to control later feedback loops. Association of P and Jak1 has been shown to prevent Jak1-STAT1 interaction^[^^55^^]^. In another mechanism of IFN antagonism, interaction of RABV P with MT cytoskeleton, TUB α/β, causes a stable binding of STAT1 with MTs, followed by the inhibited nuclear import of STAT1 and suppression of the innate immune response^[^^56^^,^^57^^]^. The interference of the RABV P with IRF-3 phosphorylation and subsequent inhibition of type I IFN induction is a well-known mechanism to manipulate the IRF-3-mediated type I IFN induction system and to evade the host immunity^[^^58^^,^^59^^]^. After the recognition of the 5′ triphosphate-ended RNA of RABV by RIG-I, a pathogen recognition receptor^[^^60^^]^, RIG-I is activated and binds to an adaptor molecule, MAVS, to signal the activation of TBK1 and IKKε. Then, IRF-3 is phosphorylated and dimerized by the activated TBK1-IKKε complex, translocates into the nucleus and induces the transcription of type I IFNs, which in turn promotes Jak-STAT signaling pathway^[^^61^^]^. A comprehensive recent study has indicated that P proteins from street RABV (HCM-9, 1088 strains), fixed RABV (CVS, Ni), and other lyssaviruses (MOKV, DUVV, LBV) could inhibit TBK-1-mediated signaling through an unknown mechanism. This inhibitory effect was followed by the prevention of type I IFN induction. Moreover, it has been demonstrated that P from only street strains of RABV (HCM-9, 1088 strains) directly interacts with IKKε and inhibits IKKε-mediated IRF-3 activation. Therefore, the functional role of P-IKKε association in the pathogenicity of RABV street strains could be the inhibition of type I IFN induction^[^^62^^]^. In another report, the direct association of RABV P (SAD L16 strain) with Rpl9 has been proposed to help RABV escaping the immune responses. Rpl9 is one of the ribosomal proteins with translational function. In the early stages of infection, P induces the translocation of Rpl9 from the nucleus to the cytoplasm and interacts with this ribosomal component. Upon L9 overexpression, RABV replication decreases significantly, and by knocking down the expression of L9, RABV replication enhances prominently, which indicates that L9 interferes with RABV replication. Of note, this effect on viral replication was not observed during *Vesicular stomatitis* virus infection, another genus of *Rhabdoviridae* family. Thus, it was suggested that in the early stages of infection, L9 binding to P disturbs the viral transcription and replication function of P, resulting in decreased RABV transcription. However, at a later stage, the amount of P exceeds the L9, which participates in RABV replication. This finding could justify the slow infectious cycle of RABV, which helps the virus to evade the host immunity^[^^63^^]^.

Incomplete autophagy is another mechanism of immune evasion, which has been induced by virulent and attenuated RABV (CVS-11, HEP-Flury strains) P and P5 isoform^[^^64^^,^^65^^]^. Autophagy is a host defense mechanism by which intracellular pathogens are removed. However, autophagy machinery is inhibited by many viruses to increase viral production^[^^66^^]^. It has been demonstrated that RABV P interacts with BECN1 and induces incomplete autophagy through activating BECN1-CASP2-AMPK-MAPK and BECN1-CASP2-AMPK-AKT-MTOR signaling pathways, which enhance the viral replication. Indeed, P-BECN1 binding decreases CASP2 expression level, which subsequently triggers the phosphorylation of AMPK. Then the phosphorylation of AKT, MAPK1/3, and MAPK11 is initiated, followed by the activation of MTOR phosphorylation via phosphorylated AKT. Autophagosome formation process is then started by phosphorylated MAPK1/3, MAPK11, and MTOR. The autophagosome, which has engulfed virions, does not fuse with the lysosomes, and lastly, virions escape degradation. It has been shown that RABV replication is prominently inhibited by BECN1 knocking down^[^^65^^]^. It has also been found that P5 is associated (stronger than P) with BECN1, thereby activating BECN1 signaling. Interestingly, this binding forms a ring-like structure that surrounds the immature autophagosome and might prevent the fusion of lysosomes and autophagosome; therefore, incomplete autophagy occurs. P5-BECN1 interaction promotes RABV self-replication^[^^64^^]^.

ABCE1 (RNase L inhibitor) is another host interactor of RABV (ERA strain) P^[^^67^^]^. This protein acts as a RNase L inhibitor to promote RNA stability^[^^68^^,^^69^^]^. ABCE1 has been shown to negatively regulate the 2-5A/RNase L antiviral pathway with viral RNA degradation activity^[^^70^^]^. Inactivation of ABCE1 inhibits RABV replication significantly, but its overexpression enhances the viral replication. Thus, this binding positively regulates virus progeny^[^^67^^]^. On the other hand, P and ABCE1 are both associated with the IFN signaling. After the production of viral RNA, RABV P tries to protect RNA from degradation by blocking the activation of IRF-3 (reviewed in^[^^21^^]^). IRF-3 activation leads to the production of IFN-α/β and stimulation of 2-5A/RNase L antiviral pathway^[^^71^^,^^72^^]^. OAS produces 2-5A from ATP. Then 2-5A dimerizes and activates RNase L, which degrades viral RNA and restricts viral replication^[^^73^^,^^74^^]^. ABCE1 inhibits RNase L activation in the mentioned signaling. It is speculated that P benefits from this ABCE1 function to protect RABV RNA from degradation^[^^67^^]^. Further studies are needed to demonstrate this issue.

IFN and ISGs are essential antiviral innate immune responses^[^^75^^]^. Upregulation of one of the mouse-specific ISGs, IIGP1, has been shown in cells and mouse brain following RABV infection, which reduced RABV (lab-attenuated CVS-B2c and wild type DRV-Mexico strains) replication and viral pathogenicity in cells and mouse model, respectively. IIGP1 deficiency increased RABV replication significantly in cells and mouse brain. Reduced pathogenicity and increased RABV replication in a mouse model, due to IIGP1 overexpression or IIGP1 knockout, were only observed through intradermal, but not intramuscular, virus inoculation. It has also been exhibited that IIGP1 exerts its effect on RABV through interaction with P and blocks its dimerization^[^^76^^]^. It seems that IIGP1-P interaction is a significant limiting molecular mechanism for the pathogenicity of attenuated RABV and surprisingly for the wild-type RABV strains, if the virus inoculated or entered intradermally. Subversion of the immune responses by lyssaviruses helps their replication and spread; therefore, it is considered as an important pathogenic mechanism in rabies infection^[^^77^^]^. Accordingly, P partners, including PML, STATs, JAK, L9, IKKε, MTs, BECN1, and ABCE1, play a prominent role in rabies pathogenesis by the innate immune suppression.

There are also some reported P-host interactions with functional roles other than immunoregulation, in rabies infection. The cytoplasmic DLC1 (LC8) is one of the first identified host partners of P in RABV (PV, CVS-11 strains) and MOKV^[^^78^^,^^79^^]^. DLC1, a component of cytoplasmic dynein motor, participates in the minus end-directed movement of cargos, along MTs^[^^80^^]^. It is supposed that P-DLC1 interaction is involved in the axonal transport of viral protein(s), along MTs in neurons^[^^79^^]^. Based on evidence, recombinant RABV without DLC1-binding motif (deleted from P) does not inhibit viral entry to the CNS of a mouse model but significantly suppresses the viral transcription (indicated by quantification of RNA transcripts by real-time PCR at the onset of infection) and replication in the CNS, consequently inhibiting the onset of RABV-induced CNS disease. This investigation proposes that DLC1-P interaction has a functional role in the regulation of viral primary transcription and is not directly involved in the retrograde axonal transport of RABV^[^^81^^]^. 

The localization of P3 isoform of P from a pathogenic strain of RABV (CVS-11) in nucleoli and binding to NCL has also been reported. NCL is a major component of the nucleoli with prominent activities in a variety of cell functions, such as ribosome biogenesis, gene expression, nucleocytoplasmic transport, and cell growth^[^^82^^]^. In this research, suppression of NCL expression using siRNA inhibited the viral protein expression and infectious virus production, indicating the importance of P3-NCL direct association in lyssavirus infection. Regarding the well-established roles of P in the modulation of host biological processes, P3 interacts with NCL to possibly facilitate its functions. The exact functional role of this binding in RABV infection cycle remains to be determined^[^^83^^]^. 

P from RABV (CVS, 8743THA, Ni, and Ni-CE strains) and ABLV, but not the other tested lyssaviruses, showed interaction with FAK to promote viral replication^[^^84^^]^. FAK, a tyrosine kinase, usually localizes at cellular focal contacts and functions in cell signaling pathways such as those involved in transcriptional regulation, cell cycle progression, and cell survival^[^^85^^,^^86^^]^. Colocalization of P with FAK was observed in NBs, which could be a strong evidence of FAK implication in viral replication. Generating a recombinant virus unable to bind with FAK exhibited the reduction of viral transcription and replication to high amount, which demonstrated that P-FAK association was necessary for viral RNA synthesis. Downregulation of FAK via RNAi also resulted in the decrease of viral protein expression. Thus, P-FAK interaction positively regulates viral replication. Whether P-FAK interaction is conserved in the genus lyssavirus remains unclear and needs further investigation^[^^84^^]^.

P protein of all lyssaviruses genotypes interacts with Cdc37/ Hsp90AA1 complex, which affects the viral life cycle^[^^87^^]^. The Hsp90 chaperone has important roles in the regulation of protein folding, maturation, and stability^[^^88^^]^and is aided by its highly specialized co-chaperone Cdc37 in maturation, stabilization, and activation of host or viral kinase targets^[^^89^^]^. During lyssavirus infection, the increased expression of Hsp90 and Cdc37 is observed. Cdc37 and Hsp90 depletion severely inhibits viral protein expression, viral RNA synthesis, and virus progeny. Interestingly, overexpression of Hsp90 and its co-chaperone just upregulates P and/or N protein, indicating the positive regulation of the P and N at protein level by this chaperone complex. It has been revealed that Cdc37/Hsp90 complex positively regulates viral infection by maintaining the stability of P, but not N. Results of that research demonstrated that Cdc37 co-chaperone helped P to load onto the Hsp90 machinery, with or without Cdc37 binding to Hsp90, thereby regulating P stability. By that study, the participation of Cdc37/Hsp90 in the stability regulation of a non-kinase target, P, was also shown^[^^87^^]^.

**Fig. 4:**
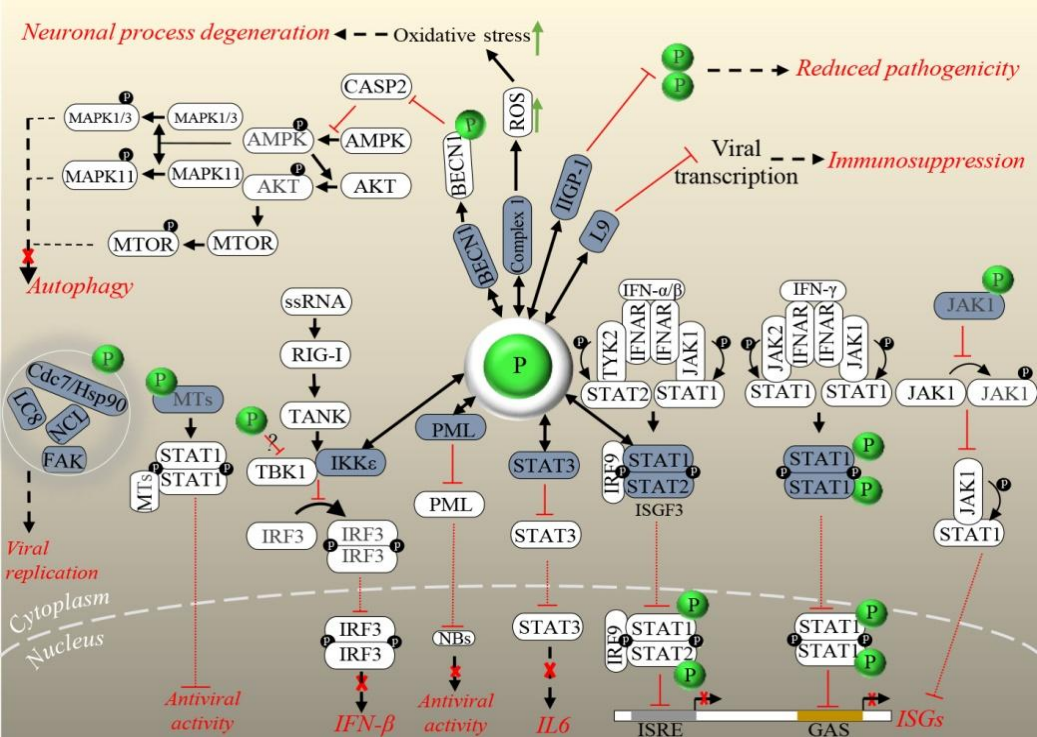
Overview of the validated physical interactions between lyssavirus P and host proteins. Host partners are colored in dark blue. Downstream signaling (if elucidated) and/or functional/pathologic outcomes of PPIs are represented. Blockage of an outcome is represented by a red cross. P in green and black circles stands for RABV phosphoprotein and a phosphate, respectively. Interaction:   , Stimulation:  , Inhibition:  , Outcome:  , Upregulation:, Translocation Inhibition: .

It is concluded that P interacting partners; DLC, NCL, FAK, and Cdc37/Hsp90 contribute to rabies infection through regulation of viral multiplication. In recent years, studies in the context of rabies pathogenesis have disclosed acute neuronal process degeneration in an experimental model of rabies, which had not been reported in the older studies. This observation is supposed to explain the severe clinical disease^[^^11^^]^. Cultured neurons infected with RABV (CVS-11) reflected axonal swelling and reduced axonal growth with the evidence of oxidative stress^[^^90^^]^. Besides, alteration of mitochondrial parameters, increased activity of mitochondrial Complex I, and subsequent increased production of ROS have been explored during rabies infection. Indeed, the increased generation of ROS due to mitochondrial dysfunction is responsible for neuronal process degeneration^[^^11^^,^^19^^]^. RABV P (CVS-11 strain) has been reported to be the inducer of mitochondrial dysfunction. P binds to mitochondrial complex I (NADH dehydrogenase), giving rise to the increased complex I activity, ROS overproduction, oxidative stress, and neuronal process degeneration, which is the pathologic outcome of this interaction^[^^91^^]^. This novel finding is postulated as a fundamental abnormality and a base of pathogenesis in rabies^[^^10^^]^. A diagram of the explained P-host PPIs has been presented in Figure 4.

*M protein*

The smallest and most abundant protein in the lyssavirus virion is multifunctional M protein^[^^92^^]^. Viral assembly/budding and regulating the balance between transcription and replication of the virus through direct or indirect interaction between L and M are the primary functions of M (Reviewed in^[^^9^^]^). However, it also acts in the downregulation of host gene expression^[^^92^^]^, apoptosis^[^^93^^]^, modulation of host innate immune defense, and virion uncoating^[^^55^^,^^94^^]^. Most of the mentioned functions for M are fulfilled through interactions with host proteins.

The PPEY core motif (amino acids 35-38) of L-domain within the M protein of RABV is suggested to implicate in efficient viral budding via interaction with WW domain of host proteins, including NEDD4. The substitution of Y with A in PPEY motif disrupts this interaction. Besides, the host-mediated ubiquitination of M is also important for RABV budding^[^^95^^]^. This binding likely relocalizes the cellular ESCRT machinery from the endosomal membrane to the plasma membrane. ESCRT machinery is composed of Tsg101, ESCRTI-III, and Vps4 components and facilitates viral budding and release^[^^96^^]^. 

It is well established that M hijacks the translational machinery of RABV-infected cells. Indeed, M interacts with eIF3h, which is involved in the regulation of the cellular translation initiation. *In vitro* translation assay has demonstrated that M suppresses the translation of mRNAs attached to ribosome via a canonical mechanism. Considering the accumulation of M in the fractions of the 40 S ribosomal subunit, M binding to eIF3-40S and formation of non-functional 40S complex and/or M binding to 48S complex and suppression of the later stages of translation are more acceptable models among proposed models for the role of M-eIF3h in translation inhibition^[^^92^^]^. 

M may also contribute to the low pathogenesis of Mokola virus (a lyssavirus of low pathogenicity) by targeting mitochondria via interaction with the terminal component of the mitochondrial respiratory chain, Cco1. M-Cco1 interaction significantly decreases Cco1 activity and ATP level of neurons, resulting in mitochondrial morphology and function disruption and subsequent apoptosis^[^^93^^]^. M also participates in the subversion of the host innate immune defense through different mechanisms. NF-kB pathway plays a key role in the regulation of the immune response to infection. After the activation of this pathway by viral infections, the transcription factors of NF-kB pathway induce the expression of antiviral cytokines^[^^97^^,^^98^^]^. Targeting the RelAp43, a member of the NF-kB family, by M of RABV (Thailand, SAD B19, and PV strains), MOKV, LBV, and EBLV-1 inhibited the expression of genes involved in immune response against viral infection, including HIAP1, IRF1, and IFN-β ^[^^94^^]^. 

**Fig. 5:**
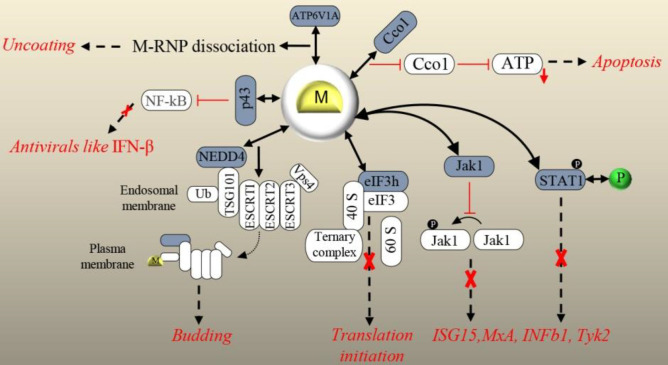
Overview of the validated physical interactions between lyssavirus M and host proteins. Host partners are colored in dark blue. Downstream signaling (if elucidated) and/or functional/pathologic outcomes of PPIs are represented. Blockage of an outcome is represented by a red cross. P in green and black circles stands for RABV phosphoprotein and a phosphate, respectively. Interaction:  , Stimulation:  , Inhibition:  , Outcome:  , Downregulation:, Translocation Stimulation: .

M not only restrains the NF-kB pathway but also cooperates with viral P to inhibit the JAK-STAT^[^^55^^]^. Activation of JAK-STAT signaling finally induces the expression of ISGs, leading to the establishment of a powerful antiviral environment inside the infected cells^[^^61^^]^. The interaction of RABV M (street isolate 8743THA) with JAK1 and STAT1 from JAK-STAT pathway has been reported by Sonthonnax *et al.*^[^^55^^]^. They proposed that M-JAK1 interaction partially inhibits JAK1 phosphorylation, which disturbs the transduction of the IFNAR signal to STAT proteins. They also suggested that M-STAT1 interaction induces the cytoplasmic retention of STAT1, therefore, enhances the capacity of RABV P to bind STAT1 and interferes with the downstream events in the pathway^[^^55^^]^. Just recently, a new function for M has been discovered in virion uncoating via binding to V-ATPase catalytic subunit A (ATP6V1A)^[^^99^^]^. V-ATPase complex is involved in endosomal acidification by pumping H^+ ^from the cell cytoplasm into the lumen. This complex is composed of two domains, including V0 endosomal membrane domain and V1 cytoplasmic domain^[^^100^^,^^101^^]^. ATP6V1A, as the catalytic subunit of V1 domain, hydrolyzes ATP to provide energy for H^+ ^pumping^[^^101^^,^^102^^]^. Overexpression and knockout of M partner showed increased and suppressed replication of RABV, respectively. The role of ATP6V1A in RABV uncoating, which in fact facilitates the virus replication, has also been reported^[^^99^^]^. After RABV entry into the host cell, the endosome-containing virus becomes acidic, and the conformation of RABV G is changed to stimulate virus-endosome membrane fusion. Then RABV M proteins dissociate and release viral nucleocapsids to cytoplasm (reviewed in^[^^9^^]^). It has been shown that in the absence of ATP6V1A, RABV uncoating does not happen, and M proteins remains associated with nucleocapsids. It has also been indicated that during membrane fusion under low pH, ATP6V1A depletion inhibits RABV uncoating. Lastly, the involvement of ATP6V1A, exactly in dissociation and separation of RABV M from nucleocapsids, has been demonstrated. The precise molecular mechanism of RABV M separation from nucleocapsids remains to be cleared^[^^99^^]^. A diagram of the explained M-host PPIs is depicted in Figure 5.

**Fig. 6.**
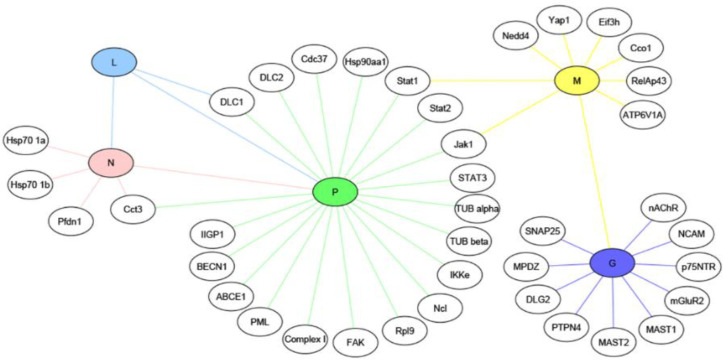
Experimentally identified and validated interactions between five proteins of lyssavirus including G (glycoprotein), N (nucleoprotein), L (RNA-dependent polymerase), P (phosphoprotein), and M (matrix protein) in colored nodes and host proteins in non-colored nodes is represented

**Fig. 7:**
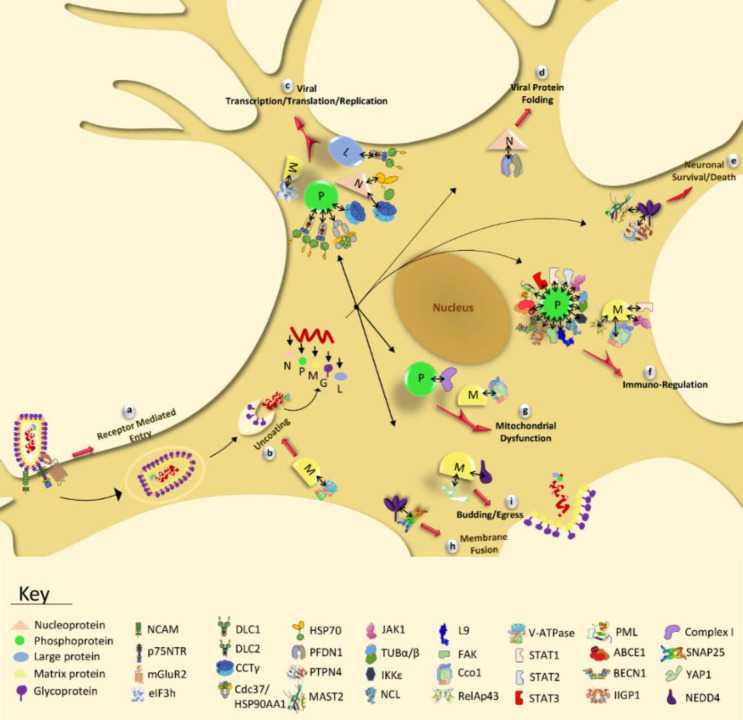
The lyssavirus-host experimentally defined PPIs and their functional/pathologic outcomes in rabies infection indicated in a neuron. G (glycoprotein), N (nucleoprotein), L (RNA-dependent polymerase or Large protein), P (phosphoprotein), and M (matrix protein) represent five viral proteins which interact with host proteins and facilitate receptor mediated entry of lyssavirus (a), uncoating (b), viral transcription/translation/replication (c), viral protein folding (d), neuronal survival/death (e), immuno-regulation (f), mitochondrial dysfunction (g), membrane fusion (h), and budding (i) during infection. Contribution of the identified PPIs in the mentioned processes has been displayed in the figure and explained in the text. The association of viral-host proteins is shown by right-leftwards arrows in black

 Taken together, M is involved in different steps of lyssavirus replication. M hijacks host cell translation machinery, thus, induces the downregulation of host genes, which normally participates in different cellular biological processes. This binding could have many pathologic outcomes for host. M also suppresses NF-kB and JAK-STAT, key antiviral pathways, and, therefore, play a role in rabies pathogenesis. Detailed information of derived lyssavirus-host physical PPIs, their relation in a network, and their involvement in different stages of viral life cycle in a neuron are shown in Table 1, Figure 6, and Figure 7, respectively.

Conclusion and future directions

Lyssaviruses, like other pathogenic viruses, take over the host cellular machinery via interacting with cellular factors for successful propagation during infection. In recent decades, a number of validated host protein partners have been introduced for lyssavirus proteins (specially for RABV) through PPI studies which are necessary for successful replication and spread of virus, and interestingly, all of these interactions are considered as potential antiviral drug targets against rabies. Many of these interactions involved in host immune suppression are central to lyssavirus pathogenesis^[^^77^^]^. Moreover, mitochondrial dysfunction (with consequent oxidative stress) is also considered as an underlying mechanism for rabies pathogenesis^[^^91^^]^. Despite these progresses, rabies is still fatal. In order to improve our understanding about rabies pathogenesis, studies on lyssavirus host PPIs should be continued to discover more and more viral partners. In fact, virus-host interactome mapping through identified PPIs could be very informative. Virus-host PIN computational analysis would reveal functional modules and protein complexes of network. These densely connected groups of proteins are involved in a specific cell function and, therefore, are functionally related^[^^103^^,^^104^^]^. Viruses strongly tend to target proteins of these modules and complexes to manipulate them and affect host signaling pathways. Disorders of signaling pathways form bases of viral pathogenesis^[^^105^^]^. Topological analysis of the PIN also allows the detection of functionally important nodes, such as hubs and bottlenecks, which could be considered as potential drug targets^[^^103^^,^^106^^]^. Viruses often target these critical nodes. Recent studies have stated that those viral targets that are host dependency factors or immune-related proteins could be used as drug targets. They should not be host essential genes, and it is better to have an altered expression in infected tissues^[^^105^^]^.

The amount of virus-host PPI data available for most viral families is still limited and is not adequate enough to generate and represent a confident PIN^[^^107^^]^. *Rhabdoviridae* family is such an example in this group^[^^107^^]^. Thus, further studies in the field of interaction proteomics should be conducted using experimental models to create a comprehensive collection of lyssavirus-host PPI data. Fortunately, in recent years the amount of data regarding lyssavirus-host physical interactions are growing fast, which is promising. Indeed, a PIN network was constructed by the integration of transcriptomic data of the CNS infected by RABV (CVS-11) and interactome data^[^^36^^]^. Analysis of this PIN demonstrated seven targeted signaling pathways, including WNT, MAPK/ERK, RAS, PI3K/AKT, toll-like receptor, JAK/STAT, and NOTCH, were involved in controlling cell cycle, cell survival, viral replication and folding, synapse regulation, and regulation of immunity. Phospholipase C, MAPK1/2, PIK3, protein kinase C, and JAK were potentially the most critical proteins in rabies pathogenesis. Integration of available transcriptomic and proteomic data with virus-host PIN contributes to model rabies infection. Investigation of this multi-dimensional PIN deciphers the most important affected biological pathways during rabies infection, suitable drug target(s), and efficient therapeutic strategies against this ancient disease.

**Table 1:** List of the prominent experimentally defined lyssavirus-host PPIs retrieved by literature and virus-host interaction databases mining

**Protein**	**Lyssavirus**	**Host protein interactor**	**Method of PPI detection and reference(s)**
G	RABV (CVS strain)	nAChR	Colocalization^[^^25^^]^, Binding inhibition assay^[^^26^^]^
			
G	RABV (CVS strain)	NCAM	Binding inhibition assay, Virus neutralization^[^^27^^]^
			
G	RABV (street strain) RABV (field strains, CVS strain & PV strain), EBLV-2	p75NTR	Screening of the cDNA library, Co-IP^[^^28^^]^ Reverse binding assay^[^^29^^]^
			
G	RABV (ERA strain, CVS-24 strain & street virus GX/09), WCBV	mGluR2	RNAi strategy, Co-IP, Pull-down assay^[^^35^^]^
			
G	RABV (Virulent strains)	MAST1,2	Yeast-two hybrid assay^[^^108^^]^
			
G	RABV (Attenuated strains)	PTPN4 MAST2 DLG2 MPDZ	Yeast-two hybrid assay^[^^108^^]^
			
G	RABV (CVS-11, SAD strains)	SNAP25	Colocalization, Co-IP^[^^37^^]^
			
N	RABV (CVS strain)	HSP70 **1A,1B**	Immunoaffinity column immobilized with anti-N^[^^39^^]^
			
N	RABV (HEP-Flury strain)	(CCTγ)	Colocalization, RNAi strategy^[^^42^^]^
			
N	RABV (HEP-Flury strain)	PFDN1	Colocalization^[^^43^^]^
			
L	RABV (SAD B19 strain)	DLC1	Colocalization, Mutation in DLC1 motif^[^^47^^]^
			
M	RABV	NEDD4	GST fusion proteins, Far-western blot assay^[^^95^^]^
			
M	RABV	YAP1	Far-western blot assay^[^^95^^]^
			
M	RABV (PV strain)	eIF3h	Yeast-two hybrid assay, Surface plasmon resonance^[^^92^^]^
			
M	MOKV	Cco1	Yeast-two hybrid assay, Co-IP, Colocalization^[^^93^^]^
			
M	RABV (Thailand, SAD B19, PV strains), MOKV, LBV, EBLV-1)	RelAp43	Yeast-two hybrid assay, Co-IP^[^^94^^]^
			
M	RABV (a street strain)	JAK1	Protein complementation assay^[^^55^^]^
			
M	RABV (a street strain)	STAT1	Protein complementation assay^[^^55^^]^
			
M	RABV (ERA strain)	ATP6V1A	Co-IP, Pull-down assay^[^^99^^]^
			
P	RABV (CVS-11 strain), MOKV	DLC1,2	Yeast-two hybrid assay^[^^79^^]^, Co-IP^[^^78^^,^^79^^]^, VirHostNet Database
			
P, P3 isoform	RABV (CVS strain)	PML	Co-IP, Colocalization^[^^50^^]^
			
P	RABV (SAD l16, CVS, SHBRV strains), MOKV, ABLV	STAT1,2	Yeast-two hybrid assay^[^^51^^]^, Co-IP^[^^51^^,^^104^^]^,VirHostNet database
			
P	RABV (CVs strain)	STAT3	Co-IP, Colocalization^[^^109^^]^
			
P (P3 Isoform)	RABV (CVSII strain)	TUB α/β	Colocalization, Biochemical test^[^^57^^]^
			
P	RABV (HEP-Flury strain)	CCTγ	Colocalization, RNAi strategy^[^^42^^]^
			
P (P3 isoform)	RABV (CVS-11strain)	NCL	Co-IP, Colocalization^[^^83^^]^
			
P	RABV (CVS, 8743THA, Ni, Ni-CE strains), ABLV	FAK	Yeast two-hybrid assay, Co-IP^[^^84^^]^
			
P	RABV (CVS-11 strain)	Mitochondrial complex I	Co-IP, Colocalization^[^^91^^]^
			
P	RABV (street strains 1088 and HCM-9)	IKKε	Co-IP^[^^62^^]^
			
P	RABV (SAD L16 strain)	Rpl9	Phage display assay, Co-IP, Pull-down assay^[^^63^^]^
			
P (P3 Isoform)	RABV (Ni strain)	MT	Colocalization, *d*STORM^[^^56^^]^
			
P	RABV (HEP-Flury, CVS-11 strains)	HSP90AA1/Cdc37	Co-IP, colocalization^[^^87^^]^
			
P	RABV (street strain 8743THA)	JAK1	Protein complementation assay^[^^55^^]^
			
P, P5 isoform	RABV (HEP-Flury, CVS-11 strains)	BECN1	Co-IP, Colocalization^[^^64^^,^^65^^]^
			
P	RABV (ERA strain)	ABCE1	Pull-down assay, Co-IP^[^^67^^]^
			
P	RABV (CVS-B2c, DRV-Mexico)	IIGP1	Co-IP, Colocalization^[^^76^^]^

## CONFLICT OF INTEREST.

None declared.

## References

[1] Rupprecht C, Kuzmin I, Meslin F (2017). Lyssaviruses and rabies: current conundrums, concerns, contradictions and controversies. F1000 research.

[2] Smith JS (1996). New aspects of rabies with emphasis on epidemiology, diagnosis, and prevention of the disease in the United States. Clinical microbiology reviews.

[3] Jackson AC (2016). Human Rabies: a 2016 Update. Current infectious disease reports.

[4] Tordo N, Kouknetzoff A (1993). The rabies virus genome: an overview. Onderstepoort journal of veterinary research.

[5] Wu X, Franka R, Velasco-Villa A, Rupprecht CE (2007). Are all lyssavirus genes equal for phylogenetic analyses?. Virus research.

[6] Kuzmin IV, Hughes GJ, Botvinkin AD, Orciari LA, Rupprecht CE (2005). Phylogenetic relationships of Irkut and West Caucasian bat viruses within the Lyssavirus genus and suggested quantitative criteria based on the N gene sequence for lyssavirus genotype definition. Virus research.

[7] Nadin-Davis SA, Anthony R, Fooks ACJ ( 2020). Molecular Epidemiology. Rabies: Scientific Basis of the Disease and Its Management.

[8] Mebatsion T, Weiland F, Conzelmann KK (1999). Matrix protein of rabies virus is responsible for the assembly and budding of bullet-shaped particles and interacts with the transmembrane spike glycoprotein G. Journal of virology.

[9] Wunner WH CK, Anthony R, Fooks ACJ (2020). Rabies virus. RABIES Scientific Basis of the Disease and Its Management. United Kingdom.

[10] Jackson AC (2016). Diabolical effects of rabies encephalitis. Journal for neurovirology.

[11] Scott CA, Rossiter JP, Andrew RD, Jackson AC (2008). Structural abnormalities in neurons are sufficient to explain the clinical disease and fatal outcome of experimental rabies in yellow fluorescent protein-expressing transgenic mice. Journal for neurovirology.

[12] Faber M, Pulmanausahakul R, Nagao K, Prosniak M, Rice AB, Koprowski H, Schnell MJ, Dietzschold B (2004). Identification of viral genomic elements responsible for rabies virus neuroinvasiveness. Proceedings of the national academy of sciences of the United States of America.

[13] Zandi F, Eslami N, Soheili M, Fayaz A, Gholami A, Vaziri B (2009). Proteomics analysis of BHK-21 cells infected with a fixed strain of rabies virus. Proteomics.

[14] Wang X, Zhang S, Sun C, Yuan ZG, Wu X, Wang D, Ding Z, Hu R (2011). Proteomic profiles of mouse neuro N2a cells infected with variant virulence of rabies viruses. Journal of microbiology and biotechnology.

[15] Thanomsridetchai N, Singhto N, Tepsumethanon V, Shuangshoti S, Wacharapluesadee S, Sinchaikul S, Chen ST, Hemachudha T, Thongboonkerd V (2011). Comprehensive proteome analysis of hippocampus, brainstem, and spinal cord from paralytic and furious dogs naturally infected with rabies. Journal of proteome research.

[16] Farahtaj F, Zandi F, Khalaj V, Biglari P, Fayaz A, Vaziri B (2013). Proteomics analysis of human brain tissue infected by street rabies virus. Molecular biology reports.

[17] Zandi F, Eslami N, Torkashvand F, Fayaz A, Khalaj V, Vaziri B (2013). Expression changes of cytoskeletal associated proteins in proteomic profiling of neuroblastoma cells infected with different strains of rabies virus. Journal of medical virology.

[18] Venugopal AK, Ghantasala SS, Selvan LD, Mahadevan A, Renuse S, Kumar P, Pawar H, Sahasrabhuddhe NA, Suja MS, Ramachandra YL, Prasad TS, Madhusudhana SN, Hc H, Chaerkady R, Satishchandra P, Pandey A, Shankar SK (2013). Quantitative proteomics for identifying biomarkers for Rabies. Clinical proteomics.

[19] Alandijany T, Kammouni W, Roy Chowdhury SK, Fernyhough P, Jackson AC (2013). Mitochondrial dysfunction in rabies virus infection of neurons. Journal of neurovirology.

[20] Gerold G, Bruening J, Weigel B, Pietschmann T (2017). Protein interactions during the flavivirus and hepacivirus life cycle. Molecular and cellular proteomics.

[21] Schnell MJ, McGettigan JP, Wirblich C, Papaneri A (2010). The cell biology of rabies virus: Using stealth to reach the brain. Nature reviews microbiology.

[22] Wiktor T, Gyorgy E, Schlumberger D, Sokol F, Koprowski H (1973). Antigenic properties of rabies virus components. Journal of immunology.

[23] Caillet-Saguy C, Maisonneuve P, Delhommel F, Terrien E, Babault N, Lafon M, Cordier F, Wolff N (2015). Strategies to interfere with PDZ-mediated interactions in neurons: What we can learn from the rabies virus. Progress in biophysics and molecular biology.

[24] Lentz TL, Burrage TG, Smith AL, Crick J, Tignor GH (1982). Is the acetylcholine receptor a rabies virus receptor?. Science.

[25] Lewis P, Fu Y, Lentz TL (2000). Rabies virus entry at the neuromuscular junction in nerve-muscle cocultures. Muscle nerve.

[26] Bracci L, Antoni G, Cusi MG, Lozzi L, Niccolai N, Petreni S, Rustici M, Santucci A, Soldani P, Valensin PE (1988). Antipeptide monoclonal antibodies inhibit the binding of rabies virus glycoprotein and alpha-bungarotoxin to the nicotinic acetylcholine receptor. Molecular immunology.

[27] Thoulouze MI, Lafage M, Schachner M, Hartmann U, Cremer H, Lafon M (1998). The neural cell adhesion molecule is a receptor for rabies virus. Journal of virology.

[28] Tuffereau C, Benejean J, Blondel D, Kieffer B, Flamand A (1998). Low-affinity nerve-growth factor receptor (P75NTR) can serve as a receptor for rabies virus. EMBO journal.

[29] Tuffereau C, Desmezieres E, Benejean J, Jallet C, Flamand A, Tordo N, Perrin P (2001). Interaction of lyssaviruses with the low-affinity nerve-growth factor receptor p75NTR. Journal of general virology.

[30] Ito N, Takayama M, Yamada K, Sugiyama M, Minamoto N (2001). Rescue of rabies virus from cloned cDNA and identification of the pathogenicity-related gene: glycoprotein gene is associated with virulence for adult mice. Journal of virology.

[31] Kucera P, Dolivo M, Coulon P, Flamand A (1985). Pathways of the early propagation of virulent and avirulent rabies strains from the eye to the brain. Journal of virology.

[32] Yan X, Mohankumar PS, Dietzschold B, Schnell MJ, Fu ZF (2002). The rabies virus glycoprotein determines the distribution of different rabies virus strains in the brain. Journal for neurovirology.

[33] Dougherty KD, Milner TA (1999). p75NTR immunoreactivity in the rat dentate gyrus is mostly within presynaptic profiles but is also found in some astrocytic and postsynaptic profiles. Journal of comparative neurology.

[34] Lafon M (2005). Rabies virus receptors. Journal of neurovirology.

[35] Wang J, Wang Z, Liu R, Shuai L, Wang X, Luo J, Wang C, Chen W, Wang X, Ge J, He X, Wen Z, Bu Z (2018). Metabotropic glutamate receptor subtype 2 is a cellular receptor for rabies virus. PLoS pathogens.

[36] Azimzadeh Jamalkandi S, Mozhgani SH, Gholami Pourbadie H, Mirzaie M, Noorbakhsh F, Vaziri B, Gholami A, Ansari-Pour N, Jafari M (2016). Systems biomedicine of rabies delineates the affected signaling pathways. Frontiers in microbiology.

[37] Yin K, Li Y, Ma Z, Yang Y, Zhao H, Liu C, Jin M, Wudong G, Sun Y, Hang T, Zhang H, Wang F, Wen Y (2020). SNAP25 regulates the release of the Rabies virus in nerve cells via SNARE complex-mediated membrane fusion. Veterinary microbiology.

[38] Masatani T, Ito N, Shimizu K, Ito Y, Nakagawa K, Sawaki Y, Koyama H, Sugiyama M (2010). Rabies virus nucleoprotein functions to evade activation of the RIG-I-mediated antiviral response. Journal of virology.

[39] Lahaye X, Vidy A, Fouquet B, Blondel D (2012). Hsp70 protein positively regulates rabies virus infection. Journal of virology.

[40] Rosenzweig R, Nillegoda NB, Mayer MP, Bukau B (2019). The Hsp70 chaperone network. Nature reviews molecularcell biology.

[41] Wan Q, Song D, Li H, He ML (2020). Stress proteins: The biological functions in virus infection, present and challenges for target-based antiviral drug development. Signal transduction and targeted therapy.

[42] Zhang J, Wu X, Zan J, Wu Y, Ye C, Ruan X, Zhou J (2013). Cellular chaperonin CCTgamma contributes to rabies virus replication during infection. Journal of virology.

[43] Zhang J, Han Q, Song Y, Chen Q, Xia X (2015). Analysis of subcellular prefoldin 1 redistribution during rabies virus infection. Jundishapur journal of microbiology.

[44] Broer L, Ikram MA, Schuur M, DeStefano AL, Bis JC, Liu F, Rivadeneira F, Uitterlinden AG, Beiser AS, Longstreth WT, Hofman A, Aulchenko Y, Seshadri S, Fitzpatrick AL, Oostra BA, Breteler MM, van Duijn CM (2011). Association of HSP70 and its co-chaperones with Alzheimer's disease. Journal of Alzheimer's disease.

[45] Morin B, Liang B, Gardner E, Ross RA, Whelan SPJ (2017). An in vitro RNA synthesis assay for rabies virus defines ribonucleoprotein interactions critical for polymerase activity. Journal of virology.

[46] Canter DM, Perrault J (1996). Stabilization of vesicular stomatitis virus L polymerase protein by P protein binding: a small deletion in the C-terminal domain of L abrogates binding. Virology.

[47] Bauer A, Nolden T, Nemitz S, Perlson E, Finke S (2015). A dynein light chain 1 binding motif in rabies virus polymerase L protein plays arole in microtubule reorganization and viral primary transcription. Journal of virology.

[48] Asthana J, Kuchibhatla A, Jana SC, Ray K, Panda D (2012). Dynein light chain 1 (LC8) association enhances microtubule stability and promotes microtubule bundling. Journal of biological chemistry.

[49] Bernardi R, Pandolfi PP (2007). Structure, dynamics and functions of promyelocytic leukaemia nuclear bodies. Nature reviews molecular cell biology.

[50] Blondel D, Regad T, Poisson N, Pavie B, Harper F, Pandolfi PP, De The H, Chelbi-Alix MK (2002). Rabies virus P and small P products interact directly with PML and reorganize PML nuclear bodies. Oncogene.

[51] Vidy A, Chelbi-Alix M, Blondel D (2005). Rabies virus P protein interacts with STAT1 and inhibits interferon signal transduction pathways. Journal of virology.

[52] Brzozka K, Finke S, Conzelmann KK (2006). Inhibition of interferon signaling by rabies virus phosphoprotein P: activation-dependent binding of STAT1 and STAT2. Journal of virology.

[53] Lieu KG, Brice A, Wiltzer L, Hirst B, Jans DA, Blondel D, Moseley GW (2013). The rabies virus interferon antagonist P protein interacts with activated STAT3 and inhibits Gp130 receptor signaling. Journal of virology.

[54] Heinrich PC, Behrmann I, Muller-Newen G, Schaper F, Graeve L (1998). Interleukin-6-type cytokine signalling through the gp130/Jak/STAT pathway. Biochemical journal.

[55] Sonthonnax F, Besson B, Bonnaud E, Jouvion G, Merino D, Larrous F, Bourhy H (2019). Lyssavirus matrix protein cooperates with phosphoprotein to modulate the Jak-Stat pathway. Scientific reports.

[56] Brice A, Whelan DR, Ito N, Shimizu K, Wiltzer-Bach L, Lo CY, Blondel D, Jans DA, Bell TD, Moseley GW (2016). Quantitative analysis of the microtubule interaction of rabies virus P3 protein: roles in immune evasion and pathogenesis. Scientific reports.

[57] Moseley GW, Lahaye X, Roth DM, Oksayan S, Filmer RP, Rowe CL, Blondel D, Jans DA (2009). Dual modes of rabies P-protein association with microtubules: a novel strategy to suppress the antiviral response. Journal of cell science.

[58] Brzozka K, Finke S, Conzelmann KK (2005). Identification of the rabies virus alpha/beta interferon antagonist: phosphoprotein P interferes with phosphorylation of interferon regulatory factor 3. Journal of virology.

[59] Rieder M, Brzozka K, Pfaller CK, Cox JH, Stitz L, Conzelmann KK (2011). Genetic dissection of interferon-antagonistic functions of rabies virus phosphoprotein: inhibition of interferon regulatory factor 3 activation is important for pathogenicity. Journal of virology.

[60] Hornung V, Ellegast J, Kim S, Brzozka K, Jung A, Kato H, Poeck H, Akira S, Conzelmann KK, Schlee M, Endres S, Hartmann G (2006). 5'-Triphosphate RNA is the ligand for RIG-I. Science.

[61] Randall RE, Goodbourn S (2008). Interferons and viruses: an interplay between induction, signalling, antiviral responses and virus countermeasures. Journal of general virology.

[62] Masatani T, Ozawa M, Yamada K, Ito N, Horie M, Matsuu A, Okuya K, Tsukiyama-Kohara K, Sugiyama M, Nishizono A (2016). Contribution of the interaction between the rabies virus P protein and I-kappa B kinase to the inhibition of type I IFN induction signalling. Journal of general virology.

[63] Li Y, Dong W, Shi Y, Deng F, Chen X, Wan C, Zhou M, Zhao L, Fu ZF, Peng G (2016). Rabies virus phosphoprotein interacts with ribosomal protein L9 and affects rabies virus replication. Virology.

[64] Liu J, Liao M, Yan Y, Yang H, Wang H, Zhou J (2020). Rabies virus phosphoprotein P5 binding to BECN1 regulates self-replication by BECN1-mediated autophagy signaling pathway. Cell communication and signaling.

[65] Liu J, Wang H, Gu J, Deng T, Yuan Z, Hu B, Xu Y, Yan Y, Zan J, Liao M, DiCaprio E, Li J, Su S, Zhou J (2017). BECN1-dependent CASP2 incomplete autophagy induction by binding to rabies virus phosphoprotein. Autophagy.

[66] Mao J, Lin E, He L, Yu J, Tan P, Zhou Y (2019). Autophagy and Viral Infection. Advances in experimental medicine and biology.

[67] Xing Liu JZ, Fang Li, Yibrah Tekle Hagoss, Weldu, Tesfagaber LW, Zilong Wang, Dongming Zhao, Zhigao, Bu Z (2020). Host protein ABCE1 interacts with the viral phosphoprotein and promotes rabies virus replication. Biosafety and Health.

[68] Le Roy F, Bisbal C, Silhol M, Martinand C, Lebleu B, Salehzada T (2001). The 2-5A/RNase L/RNase L inhibitor (RLI) [correction of (RNI)] pathway regulates mitochondrial mRNAs stability in interferon alpha-treated H9 cells. Journal of biological chemistry.

[69] Tian Y, Han X, Tian DL (2012). The biological regulation of ABCE1. IUBMB Life.

[70] Bisbal C, Salehzada T, Silhol M, Martinand C, Le Roy F, Lebleu B (2001). The 2-5A/RNase L pathway and inhibition by RNase L inhibitor (RLI). Methods in molecular biology.

[71] Chen S, Zhang W, Wu Z, Zhang J, Wang M, Jia R, Zhu D, Liu M, Sun K, Yang Q, Wu Y, Chen X, Cheng A (2017). Goose Mx and OASL play vital roles in the antiviral effects of type I, II, and III interferon against newly emerging avian flavivirus. Frontiers in immunology.

[72] Melchjorsen J, Kristiansen H, Christiansen R, Rintahaka J, Matikainen S, Paludan SR, Hartmann R (2009). Differential regulation of the OASL and OAS1 genes in response to viral infections. Journal of interferon and cytokine research.

[73] Silverman RH (2007). Viral encounters with 2',5'-oligoadenylate synthetase and RNase L during the interferon antiviral response. Journal of virology.

[74] Zhu J, Ghosh A, Sarkar SN (2015). OASL-a new player in controlling antiviral innate immunity. Currrent opinion virology.

[75] Schoggins JW, Rice CM (2011). Interferon-stimulated genes and their antiviral effector functions. Currrent opinion virology.

[76] Tian B, Yuan Y, Yang Y, Luo Z, Sui B, Zhou M, Fu ZF, Zhao L (2020). Interferon-inducible GTPase 1 impedes the dimerization of rabies virus phosphoprotein and restricts viral replication. Journal of virology.

[77] Scott TP, Nel LH (2016). Subversion of the Immune Response by Rabies Virus. Viruses.

[78] Jacob Y, Badrane H, Ceccaldi PE, Tordo N (2000). Cytoplasmic dynein LC8 interacts with lyssavirus phosphoprotein. Journal of virology.

[79] Raux H, Flamand A, Blondel D (2000). Interaction of the rabies virus P protein with the LC8 dynein light chain. Journal of virology.

[80] Reck-Peterson SL, Redwine WB, Vale RD, Carter AP (2018). The cytoplasmic dynein transport machinery and its many cargoes. Nature reviews molecular cell biology.

[81] Tan GS, Preuss MA, Williams JC, Schnell MJ (2007). The dynein light chain 8 binding motif of rabies virus phosphoprotein promotes efficient viral transcription. Proceedings of the national academy of sciences of the United States of America.

[82] Salvetti A, Coute Y, Epstein A, Arata L, Kraut A, Navratil V, Bouvet P, Greco A (2016). Nuclear functions of nucleolin through global proteomics and interactomic approaches. Journal of proteome research.

[83] Oksayan S, Nikolic J, David CT, Blondel D, Jans DA, Moseley GW (2015). Identification of a role for nucleolin in rabies virus infection. Journal of virology.

[84] Fouquet B, Nikolic J, Larrous F, Bourhy H, Wirblich C, Lagaudriere-Gesbert C, Blondel D (2015). Focal adhesion kinase is involved in rabies virus infection through its interaction with viral phosphoprotein P. Journal of virology.

[85] Ilic D, Damsky CH, Yamamoto T (1997). Focal adhesion kinase: at the crossroads of signal transduction. Journal of cell science.

[86] Parsons JT (2003). Focal adhesion kinase: the first ten years. Journal of cell science.

[87] Xu Y, Liu F, Liu J, Wang D, Yan Y, Ji S, Zan J, Zhou J (2016). The co-chaperone Cdc37 regulates the rabies virus phosphoprotein stability by targeting to Hsp90AA1 machinery. Scientific reports.

[88] Taipale M, Jarosz DF, Lindquist S (2010). HSP90 at the hub of protein homeostasis: emerging mechanistic insights. Nature reviews molecularcell biology.

[89] Stepanova L, Leng X, Parker SB, Harper JW (1996). Mammalian p50Cdc37 is a protein kinase-targeting subunit of Hsp90 that binds and stabilizes Cdk4. Genes and development.

[90] Jackson AC, Kammouni W, Zherebitskaya E, Fernyhough P (2010). Role of oxidative stress in rabies virus infection of adult mouse dorsal root ganglion neurons. Journal of virology.

[91] Kammouni W, Wood H, Saleh A, Appolinario CM, Fernyhough P, Jackson AC (2015). Rabies virus phosphoprotein interacts with mitochondrial Complex I and induces mitochondrial dysfunction and oxidative stress. Journal for neurovirology.

[92] Komarova AV, Real E, Borman AM, Brocard M, England P, Tordo N, Hershey JW, Kean KM, Jacob Y (2007). Rabies virus matrix protein interplay with eIF3, new insights into rabies virus pathogenesis. Nucleic acids research.

[93] Gholami A, Kassis R, Real E, Delmas O, Guadagnini S, Larrous F, Obach D, Prevost MC, Jacob Y, Bourhy H (2008). Mitochondrial dysfunction in lyssavirus-induced apoptosis. Journal of virology.

[94] Luco S, Delmas O, Vidalain PO, Tangy F, Weil R, Bourhy H (2012). RelAp43, a member of the NF-kappaB family involved in innate immune response against Lyssavirus infection. PLoS Pathog.

[95] Harty RN, Paragas J, Sudol M, Palese P (1999). A proline-rich motif within the matrix protein of vesicular stomatitis virus and rabies virus interacts with WW domains of cellular proteins: implications for viral budding. Journal of virology.

[96] Jayakar HR, Jeetendra E, Whitt MA (2004). Rhabdovirus assembly and budding. Virus research.

[97] Versteeg GA, Garcia-Sastre A (2010). Viral tricks to grid-lock the type I interferon system. Current opinion in microbioogy.

[98] Sadler AJ, Williams BR (2008). Interferon-inducible antiviral effectors. Nature reviews immunology.

[99] Liu X, Li F, Zhang J, Wang L, Wang J, Wen Z, Wang Z, Shuai L, Wang X, Ge J, Zhao D, Bu Z (2020). The ATPase ATP6V1A facilitates rabies virus replication by promoting virion uncoating and interacting with the viral matrix protein. Journal of biological chemistry.

[100] Wang C, Zhao T, Li Y, Huang G, White MA, Gao J (2017). Investigation of endosome and lysosome biology by ultra pH-sensitive nanoprobes. Advanced drug delivery reviews.

[101] Breton S, Brown D (2013). Regulation of luminal acidification by the V-ATPase. Physiology (Bethesda).

[102] Forgac M (2007). Vacuolar ATPases: rotary proton pumps in physiology and pathophysiology. Nature reviews molecularcell biology.

[103] De Las Rivas J (2012). Protein-protein interaction networks: unraveling the wiring of molecular Fontanillo C machines within the cell. Briefings in functional genomics.

[104] Mukhopadhyay A, Maulik U (2014). Network-based study reveals potential infection pathways of hepatitis-C leading to various diseases. PLoS one.

[105] Yang S, Fu C, Lian X, Dong X, Zhang Z (2019). Understanding human-virus protein-protein interactions using a human protein complex-based analysis framework. mSystems.

[106] Watanabe T, Kawakami E, Shoemaker JE, Lopes TJ, Matsuoka Y, Tomita Y, Kozuka-Hata H, Gorai T, Kuwahara T, Takeda E, Nagata A, Takano R, Kiso M, Yamashita M, Sakai-Tagawa Y, Katsura H, Nonaka N, Fujii H, Fujii K, Sugita Y, Noda T, Goto H, Fukuyama S, Watanabe S, Neumann G, Oyama M, Kitano H, Kawaoka Y (2014). Influenza virus-host interactome screen as a platform for antiviral drug development. Cell host and microbe.

[107] Brito AF, Pinney JW (2017). Protein-Protein Interactions in Virus-Host Systems. Frontiers in microbiology.

[108] Terrien E, Chaffotte A, Lafage M, Khan Z, Prehaud C, Cordier F, Simenel C, Delepierre M, Buc H, Lafon M, Wolff N (2012). Interference with the PTEN-MAST2 interaction by a viral protein leads to cellular relocalization of PTEN. Science signaling.

[109] Wiltzer L, Larrous F, Oksayan S, Ito N, Marsh GA, Wang LF, Blondel D, Bourhy H, Jans DA, Moseley GW (2012). Conservation of a unique mechanism of immune evasion across the Lyssavirus genus. Journal of virology.

